# CE Accreditation and Barriers to CE Marking of Pediatric Drug Calculators for Mobile Devices: Scoping Review and Qualitative Analysis

**DOI:** 10.2196/31333

**Published:** 2021-12-13

**Authors:** Charlotte Koldeweij, Jonathan Clarke, Joppe Nijman, Calandra Feather, Saskia N de Wildt, Nicholas Appelbaum

**Affiliations:** 1 Institute of Global Health Innovation Imperial College London London United Kingdom; 2 Department of Pharmacology and Toxicology Radboud Institute for Health Sciences Radboud University Medical Center Nijmegen Netherlands; 3 Centre for Mathematics of Precision Healthcare Imperial College London London United Kingdom; 4 Department of Pediatric Intensive Care University Medical Centre Utrecht Utrecht Netherlands; 5 Intensive Care and Department of Intensive Care Erasmus MC Sophia Children’s Hospital Rotterdam Netherlands

**Keywords:** pediatric, drug dosage calculator, European regulations, safety, medical devices, medical errors, app, application, mobile health, pharmacy

## Abstract

**Background:**

Pediatric drug calculators (PDCs) intended for clinical use qualify as medical devices under the Medical Device Directive and the Medical Device Regulation. The extent to which they comply with European standards on quality and safety is unknown.

**Objective:**

This study determines the number of PDCs available as mobile apps for use in the Netherlands that bear a CE mark, and explore the factors influencing the CE marking of such devices among app developers.

**Methods:**

A scoping review of Google Play Store and Apple App Store was conducted to identify PDCs available for download in the Netherlands. CE accreditation of the sampled apps was determined by consulting the app landing pages on app stores, by screening the United Kingdom Medicines and Healthcare products Regulatory Agency’s online registry of medical devices, and by surveying app developers. The barriers to CE accreditation were also explored through a survey of app developers.

**Results:**

Of 632 screened apps, 74 were eligible, including 60 pediatric drug dosage calculators and 14 infusion rate calculators. One app was CE marked. Of the 20 (34%) respondents to the survey, 8 considered their apps not to be medical devices based on their intent of use or functionality. Three developers had not aimed to make their app available for use in Europe. Other barriers that may explain the limited CE accreditation of sampled PDC apps included poor awareness of European regulations among developers and a lack of restrictions when placing PDCs in app stores.

**Conclusions:**

The compliance of PDCs with European standards on medical devices is poor. This puts clinicians and their patients at risk of medical errors resulting from the largely unrestricted use of these apps.

## Introduction

The use of mobile health (mHealth) apps among clinicians is growing [1,2]. In 2015, 60% of medical doctors in the Netherlands used at least 1 mHealth app [3]. The widespread use of mHealth apps creates new risks for patient safety [4,5]. These risks include both technical malfunctions and misuse, either of which may lead to life-threatening medical errors [4].

To mitigate these risks, the European Union (EU)’s 2007 Medical Device Directive (93/42/ECC) (MDD) qualifies “any…software…intended by the manufacturer to be used…for the purpose of diagnosis…or treatment…of disease” as a medical device [6]. The MDD categorizes medical devices into 4 classes of risk (Classes I, IIa, IIb, and III) based on their technical characteristics, invasiveness, and potential for harm. Each class of risk determines a specific conformity assessment procedure for legally entering the European market. The higher the class of risk, the more stringent the conformity assessment procedure, with the overall objective being to provide adequate safeguards for users to be able to safely use the device. For example, for a Class II medical device, conformity assessment entails an evaluation of the device’s technical documentation as well as its quality management system [6]. Depending on the device classification, conformity assessment is performed by either the manufacturer (Class I) or a European Notified Body (Class IIa and above). Once the conformity assessment is complete, medical devices obtain a CE mark, indicating their conformity with European health and safety standards, allowing them to be made available to the public within the extended single market of the European Economic Area (EEA) [7].

In May 2017, the MDD was replaced by the Medical Device Regulation (2017/745) (MDR) [8]. Among other changes, the MDR addresses software as a distinct item and establishes more stringent classification rules for software apps under Rule 11 [8,9]. By May 2021, all new devices placed on the European market were required to comply with the MDR. Devices already certified under the MDD may continue to be placed on the European market until May 2024, with the exception of Class I devices receiving a higher class under the MDR [10,11].

Despite increasingly binding European regulations, poor compliance of mHealth apps with EU certification requirements has been found. An examination of a sample of health apps freely available on several app stores by the Dutch Royal Institute for Public Health and the Environment reported that less than half are CE marked, as appropriate [12].

Pediatric drug calculators (PDCs) are tools designed to help clinicians overcome the complexities of dosing calculations in pediatrics and are increasingly used in clinical care [13]. By allowing clinicians to calculate drug doses to be administered to children based on patient characteristics, most often their weight, PDCs constitute 1 example of medical apps potentially associated with new risks for patients [14,15]. PDCs have received little scrutiny with regard to their conformity to European standards [12,16]. In this study, we perform a scoping review of Google Play Store and Apple App Store to identify PDCs available for download in the Netherlands and determine their CE accreditation status. Barriers to CE accreditation are explored through developer surveys and interviews.

## Methods

### Definitions

In this study, a PDC was defined as a mobile app that allows clinicians to enter information about an individual child’s weight or age in order to calculate a recommended drug dosage for that child. Programs designed to determine an infusion rate or dilution volume for a given drug dosage were also defined as PDCs.

Because PDCs perform transformation of data intended to inform treatment decisions for individual patients, they would qualify as medical devices under the MDD and the MDR [6,8,17,18]. According to the MDD, PDCs would be classified as Class I medical devices [6]. In line with Rule 11 of the MDR, any software “intended to provide information…used to take decisions with…therapeutic purposes” falls under Class IIa. When such decisions can cause “a serious deterioration of a person's state of health…,” the software falls under Class IIb [8]. If the decision has the potential to “cause death or an irreversible deterioration of a person's state of health,” the software receives a class III classification [8]. PDCs intended for clinical use would therefore be classified as Class IIa or above under the MDR.

The terms “application provider,” “manufacturer,” and “developer” have been used interchangeably in this study.

### Search Strategy and Screening

A scoping review of PDCs available on app stores was performed using the Preferred Reporting Items for Systematic Reviews and Meta-Analyses (PRISMA) methodology [19]. Apps were searched for on Google Play Store (Android system, desktop version) and Apple App Store (iOS system, mobile version) between April 8 and April 19, 2020. Separate searches were performed in both app stores using the following search terms: “pediatric drug,” “pediatric drug calculator,” “neonatal drug,” and “neonatal drug calculator*.”* Sample searches for the terms “pediatric drug” and “pediatric drug calculator” were also conducted in both app stores. They produced identical results to the ones obtained for the previous search terms and were hence not completed. All sampled apps were deduplicated and screened by an individual reviewer (author CK). The availability of each app on Google Play Store and Apple App Store was verified independently of the initial search results.

Eligibility criteria were defined a priori*.* Apps were required to appear to be designed for health care professionals, including medical students, doctors, nurses, and paramedics. A PDC was required to be the main functionality or 1 of several functionalities of each app. The drug dosage calculator should have been developed for a pediatric population, with users able to calculate a drug dose for a specific weight, age, or body surface area. PDCs for oral or intravenous drugs were eligible if they covered more than 1 drug. Infusion dilution and infusion rate calculators were also included. Apps solely performing calculations for parenteral nutrition, maintenance fluids, electrolytes, or chemotherapy were excluded.

PDCs were screened based on their name, description, and screenshots available in each app store. Only apps that were freely available were downloaded.

### Data Extraction and Qualitative Analysis

The name, manufacturer, and country of manufacture of each PDC were collected. Information about the type of calculations performed (drug dosage or infusion dilution or rate), the intended location of use (within or outside the EEA), and the number of downloads on Google Play Store were captured. To determine the CE marking status, we searched the PDC description and screenshots in app stores, any documentation provided on the app website, and relevant pages of the downloaded app (License, Disclaimer, About, or Terms and Conditions).

All PDC manufacturers with identifiable contact information were contacted through email (see Multimedia Appendix 1). Developers were invited to provide information about the type of calculations performed and the intended location of use of their PDC. They were asked whether their apps were CE marked and were invited to describe their considerations in choosing whether to pursue CE marking. They were additionally asked to report any barriers encountered in the CE accreditation process. When their responses called for clarification, they were recontacted. App providers were interviewed through video calls whenever they accepted to do so.

Data obtained from PDC manufacturers were anonymized through the attribution of a numeric code and access restricted to the first author. Responses from developers were manually analyzed. Separate considerations and barriers to CE accreditation were identified from their responses and categorized through thematic inductive analysis by 1 reviewer (CK). Developers’ responses were coded against the identified themes. The coded list of barriers and considerations was discussed with 2 additional authors (JC and NA), and discrepancies were resolved through consensus.

When relevant information about CE accreditation could not be obtained from the aforementioned sources, registration of the app on the United Kingdom Medicines and Healthcare products Regulatory Agency (MHRA) was searched using the MHRA website’s search function with the app manufacturer name [18]. At the time of data collection, the MHRA website constituted the only available repository of information related to the CE accreditation of medical devices in the EU.

Both app stores were contacted through their online contact pages to inquire about their review process for medical apps and the extent of their collaboration with European regulatory authorities.

### Ethics

According to the Dutch Medical Research Involving Human Subjects Act, formal ethical review was not needed. Interviewees provided informed consent through email to collect and store their anonymized responses and for these to be published. Patient consent was not applicable.

### Data Sharing

All data that informed this study are contained within the article and its supplementary files.

### Public and Patient Involvement

Patients and the public were not involved in the design, conduct, reporting, dissemination plans of this research.

### Transparency

The lead author affirms that this manuscript is an honest, accurate, and transparent account of the study being reported; that no important aspects of the study have been omitted; and that any discrepancies from the study, as planned, have been explained.

## Results

### Inclusion and Classification of Apps

**Figure 1:**
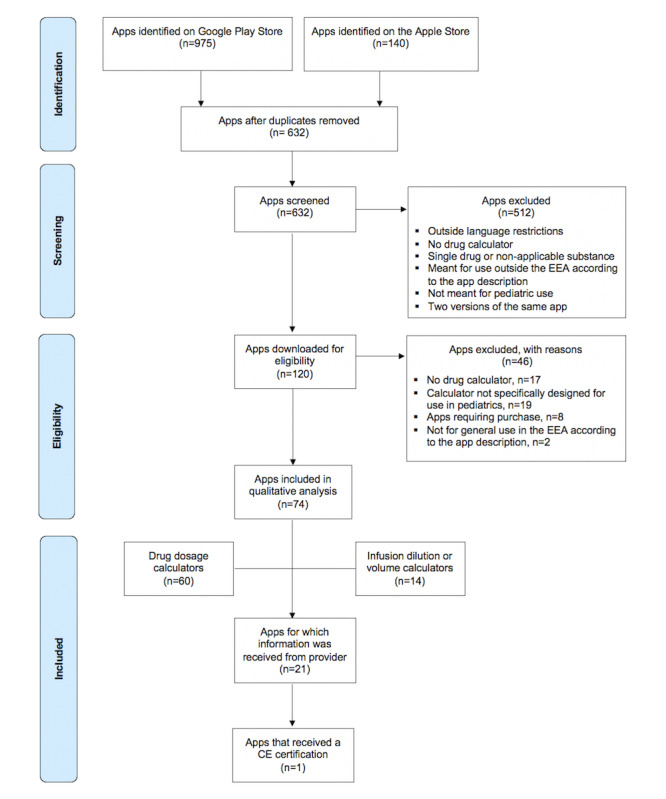
Flowchart. EEA: European Economic Area.

A total of 632 PDCs were included for screening after deduplication (see Figure 1 and Multimedia Appendix 2). Of these, 74 (11.7%) PDCs met the inclusion criteria: 66 of the 74 (89.2%) apps were available on Google Play Store and 8 (10.8%) on Apple App Store (see Table 1); 20 (27%) apps were available on both stores. In addition, 18 of 74 (24.3%) apps were developed in EEA countries, 60 (81.1%) included a drug dosage calculator, and 14 (18.9%) incorporated an infusion rate or infusion dilution calculator without a drug dosage calculator. The number of installations per app on Google Play Store varied from 10-100 to over 100,000; 13 of 74 (17.6%) apps had been installed over 100,000 times. Of the 74 screened PDCs, only 1 (1.4%) app was CE marked.

**Table 1:** Sampled app characteristics, including CE accreditation.

Number	App name	Country	App store	Last update^a^	Installations (n)^b^	Purchasing fee	CE marking
1	AnestCRITIC Crisis y Anestesia	Spain	Google Play	2019	5000-10,000	No	No
2	Anesthesia Assist	Portugal	Google Play	2019	50,000-100,000	No	No
3	Anesthesia Drugs Fast	United States	Google Play	2018	100-1000	Yes	No
4	Anesthesia ICC infusion calculator	Spain	Google Play	2019	5000-10,000	No	No^c^
5	Anesthesiologist	United States	Google Play	2016	Over 100,000	No	No^c^
6	Anesthetic drugs	India	Google Play	2019	5000-10,000	No	No
7	Clinical Calculator PLUS	United States	Google Play	2020	1000-5000	Yes	No
8	CoPE Paediatric Emergency	Denmark	Google Play	2019	1000-5000	Yes	No
9	Dosage Calculator	Hong Kong	Google Play	2019	Over 100,000	Yes	No
10	Dose calculator	Egypt	Google Play	2020	Over 100,000	No	No
11	Dosefinder 1	United Kingdom	Google Play	2016	5000-10,000	No	No^c^
12	Dosis Pediatricas	—^d^	Google Play	2019	1000-5000	No	No
13	DosisPedia	Spain	Google Play, Apple App	2020	Over 100,000	No	No^c^
14	DrDrugs: Drug Guide for Physicians - 2020 Updates	United States	Google Play	2020	1000-5000	Yes	No
15	Drug dosage calculations	Saudi Arabia	Google Play	2018	50,000-100,000	No	No^c^
16	Drug Dose	Ukraine	Google Play	2016	5000-10,000	No	No
17	DrugCalc: Pediatric dosing calculator	Thailand	Google Play	2017	5000-10,000	No	No^c^
18	DrugDoses	United States	Google Play, Apple App	2019	5000-10,000	Yes	No^c^
19	Drugscape dose calculator	Jordan	Google Play	2019	10,000-50,000	No	No
20	Easy Drug Dose Calculator	Australia	Google Play	2018	Over 100,000	No	No
21	EBMcalc Pediatrics	United States	Apple App	—	—	Yes	No^c^
22	eBroselow SafeDose	United States	Google Play, Apple App	2020	Over 100,000	No	No
23	EMS Calculator	United States	Apple App	—	—	Yes	No
24	EMS Drugs Fast	United States	Apple App	—	—	Yes	No
25	EnfermerApp	Chile	Google Play	2019	10,000-50,000	No	No^c^
26	GIR Calc	United States	Apple App	—	—	No	No
27	Infinite dose: the smart dosage calculator	Egypt	Google Play	2018	10,000-50,000	No	No^c^
28	Infusions	Colombia	Google Play, Apple App	2020	Over 100,000	No	No
29	Infusions - Infusions Calculator	Egypt	Google Play	2019	10,000-50,000	No	No^c^
30	Inotropes Rate Calculator	Jordan	Google Play	2016	5000-10,000	No	No^c^
31	Intravenous Medications Gahart	United States	Google Play	2019	5000-10,000	Yes	No^c^
32	Kids Drug Dosage Calc - PaedRx	India	Google Play	2013	50,000-100,000	No	No
33	Lexicomp	United States	Google Play, Apple App	2020	Over 100,000	Yes	No^c^
34	Medic Dose Calculator	India	Google Play	2018	5000-10,000	No	No^c^
35	Medical Calculator	United States	Google Play	2019	500-1000	No	No^c^
36	Mediquations Medical Calculator	United States	Google Play, Apple App	2018	10,000-50,000	Yes	No
37	Millidos: Pediatric Drug Dosages	Syria	Google Play	2019	10,000-50,000	No	No
38	MKD Dosage Calc	—	Google Play	2019	100-1000	No	No
39	Neomate	United Kingdom	Google Play, Apple App	2017	50,000-100,000	No	Yes
40	NeonaCal	Ireland	Apple App	—	—	Yes	No
41	Neonatology	United Kingdom	Google Play, Apple App	2019	100-1000	Yes	No
42	NICU	United Kingdom	Google Play, Apple App	2019	100-1000	Yes	No
43	Nursing calculator	India	Google Play	2020	Over 100,000	No	No^c^
44	Paediatric Emergencies	United Kingdom	Google Play	2019	1000-5000	No	No
45	Paediatric Emergency Tools	United Kingdom	Google Play, Apple App	2019	100-1000	Yes	No
46	palmPEDi: Pediatric Tape	United States	Google Play, Apple App	2013	5000-10,000	Yes	No
47	Paramedic Meds	United States	Google Play	2019	10,000-50,000	Yes	No
48	PedAMINES	Switzerland	Google Play, Apple App	2018	10-50	Yes	No
49	Ped(z) - Pediatric Calculator	Germany	Google Play, Apple App	2017	Over 100,000	No	No^c^
50	PedCalc	Egypt	Google Play	2017	10,000-50,000	No	No^c^
51	Pedi Crisis 2.0	United States	Google Play, Apple App	2019	1000-10,000	No	No
52	Pedi Help	Switzerland	Google Play, Apple App	2017	50,000-10,000	No	No
53	Pedi Safe Medications	United States	Google Play	2016	10,000-50,000	Yes	No
54	Pedi Safe Pediatric Anesthesia	United States	Apple App	—	—	No	No
55	Pedi STAT	Canada	Google Play, Apple App	2018	Over 100,000	Yes	No
56	Pediatria calculadora dosis/kg	—	Google Play	2020	Over 100,000	Yes	No
57	Pediatric dosage calculator	Hong Kong	Google Play	2019	Over 100,000	No	No
58	Pediatric dose calculator	Netherlands	Google Play, Apple App	2016	50-100	Yes	No
59	Pediatric doses calculator	Egypt	Google Play	2020	10,000-50,000	No	No
60	Pediatric Gas for Anesthesia	United States	Apple App	—	—	Yes	No
61	Pediatric Guideline/Emergency/Pediatric child care	United Kingdom	Google Play	2020	Over 100,000	No	No
62	Pediatric IV calculator	Netherlands	Google Play, Apple App	2016	10-50	Yes	No
63	Pediatric IV dosage	—	Google Play	2014	50,000-100,000	No	No
64	Pediatric IV Rate	United States	Google Play	2019	50-100	No	No^c^
65	Pediatric oral dosage	—	Google Play	2015	50,000-100,000	No	No
66	Pediatric pedia	Middle East	Apple App	—	—	No	No
67	PediRef: Pocket Pediatrics	United States	Google Play	2017	10,000-50,000	No	No
68	PedsGuide	United States	Google Play, Apple App	2019	1000-5000	No	No
69	PeKemecum	Spain	Google Play	2019	50,000-100,000	No	No
70	PICU Calculator	United Kingdom	Google Play, Apple App	2019	1000-5000	No	No
71	PICUDoctor 5 - Cardiac Guide	Australia	Google Play	2015	10,000-50,000	Yes	No^c^
72	RightDose	United States	Apple App	—	—	No	No
73	SmartPedi-Pediatric Treatment & Dose Calculator	Bangladesh	Google Play	2019	5000-10,000	No	No
74	UCIN-Calc Beta	Dominican Republic	Google Play	2018	5000-10,000	No	No

^a^Last update on Google Play Store.

^b^Number of installs on Google Play Store on May 8, 2020.

^c^Information obtained from the app developer.

^d^Not available.

### Qualitative Analysis

#### App Developers

Of 61 app developers, 59 (96.7%) for whom contact information was available were contacted through email; 1 (1.6%) developer was additionally contacted through a video call. Responses were obtained from 20 of 59 (33.9%) providers that developed 21 apps (see Table 2). Of the 20 developers, 3 (15%) were based in the EEA. None of the apps developed by the respondents were CE marked. In addition, 2 of the 20 developers (10%) indicated that they understood that their apps qualified as Class I medical devices under the MDD but were not CE marked (developers 4 and 8), while 2 (10%) had attempted to get their apps CE marked but were unsuccessful (developers 5 and 18).

The most frequent reason for not pursuing CE accreditation provided by developers was that in their view, their apps did not qualify as medical devices (8/20, 40%). Various arguments informed this assessment. Of the 20 developers, 2 (10%) referred to the intended use of their apps, stating that the apps were designed as a *reference* or an *educational* tool for clinicians as opposed to a clinical decision-making aid (developers 11 and 15). This disclaimer was also frequently provided in the end-user licenses of sampled apps. Other developers referred to their apps’ functionality, describing them as *digital documents* (developer 1) or *books* (developer 14), which did not entail manipulation of data. In both cases, the functionality of the apps involved transformation of data. Arguments pertaining to functionality also examined the nature of the information being input into and delivered by a given app, and the weight of the result in determining the process of care. Developer 18 highlighted a difference between drug dosage calculators that could be seen as medical devices owing to their recommending a specific drug dose based on an individual patient’s characteristics, and the infusion rate or dilution calculators performing simple conversion operations on pre-established drug prescriptions. According to developers 8 and 13, the level of transparency and complexity of the computations performed by an app constituted key factors when determining whether it qualified as a medical device. Developer 13 suggested that if the calculations performed by an app are simple enough to be immediately replicable by users, then the app would not qualify as a medical device. Developer 8 suggested that even for more complex calculations, if the calculations are linked to user-accessible formulae and bibliographic support, the app should not be classified as a medical device.

Other reasons put forward by developers as justification for not CE-marking their apps included a lack of knowledge of European legislation on medical devices (3/20, 15%), the fact that their apps were not devised for use in EEA countries (3/20, 15%), and the fact that no certification was required for access to Google Play or Apple App Store (4/20, 20%). Several manufacturers described app stores as implicit arbiters for matters of regulatory compliance or safety (“My app was evaluated in the…store by the public user” or “It was very easy to place on the…store.”). Of the 20 providers, 3 (15%) indicated that Apple App Store was more restrictive than Google Play Store when granting access for PDCs; 1 (5%) developer outside the EEA argued that his app did not require testing or accreditation according to the regulations of his country (developer 15).

Several barriers to CE marking were outlined by developers. Of the 20 manufacturers, 2 (10%) indicated that the process was too complex (developers 4 and 18), and 1 (5%) said it was too costly to take on as an individual developer or a small enterprise (developer 18). This appeared more generally relevant across the sample, with multiple developers stating that they were clinicians with programming skills who developed a PDC “as a hobby” (developer 6) or “for their own use” (developer 16). An added barrier in this view concerned the lack of institutional support received by app manufacturers seeking to obtain a CE marking that were also affiliated to a hospital or a university. After receiving confirmation from national regulatory authorities that his app qualified as a Class I medical device under the MDD, developer 4 asked the relevant national health care institution for its support in the CE accreditation process. He did not obtain this support due to the institution’s concerns over the costs and associated legal liability. He shared that “developers are often left unsupported by their associated institutions...I think mostly because of a lack of experience and knowledge regarding the governance and legal implications, many institutions feel vulnerable and unwilling to engage with regulatory bodies.” Overall, this “had an unfortunate regressive effect” on the dissemination of his app. Independently of CE accreditation, 5 of 20 (25%) developers had sought alternative forms of clinical validation for their apps, for example, by national experts.

**Table 2:** Developer responses on the barriers to CE accreditation.

Barriers to CE accreditation and other considerations outlined by developers on the CE accreditation process	Developer^a^																					Total developers (n)
	1	2	3	4	5	6	7	8	9	10	11	12	13	14	15	16	17	18	19	20		
No reason provided	✗^b^	✓^c^	✗	✗	✗	✓	✗	✗	✗	✓	✗	✓	✗	✗	✗	✗	✗	✗	✓	✗	5	
App not meant for use in European countries	✗	✗	✓	✗	✗	✗	✓	✗	✗	✗	✗	✗	✗	✗	✗	✓	✗	✗	✗	✗	3	
Not a medical device	✓	✗	✗	✗	✗	✗	✗	✓	✓	✗	✓	✗	✗	✓	✓	✗	✗	✓	✗	✓	8	
Unaware of the EEA^d^ medical device regulations	✗	✗	✓	✗	✓	✗	✓	✗	✗	✗	✗	✗	✗	✗	✗	✗	✗	✗	✗	✗	3	
Compliant with national regulations (non-EEA)	✗	✗	✗	✗	✗	✗	✗	✗	✗	✗	✗	✗	✗	✗	✓	✗	✗	✗	✗	✗	1	
App store not requiring certification	✗	✗	✗	✗	✗	✗	✗	✗	✓	✗	✗	✗	✓	✗	✗	✗	✓	✗	✗	✗	3	
Discussed with national certification authorities	✗	✗	✗	✓	✗	✗	✗	✗	✗	✗	✗	✗	✗	✗	✗	✗	✗	✗	✗	✗	1	
Did not receive institutional support	✗	✗	✗	✓	✗	✗	✗	✗	✗	✗	✗	✗	✗	✗	✗	✗	✗	✗	✗	✗	1	
Process too complex	✗	✗	✗	✓	✗	✗	✗	✗	✗	✗	✗	✗	✗	✗	✗	✗	✗	✓	✗	✗	2	
Process too costly	✗	✗	✗	✗	✗	✗	✗	✗	✗	✗	✗	✗	✗	✗	✗	✗	✗	✓	✗	✗	1	
App undergoing another form of validation	✓	✗	✗	✓	✓	✗	✗	✓	✗	✗	✗	✗	✗	✗	✗	✗	✗	✓	✗	✗	5	

^a^Of the 20 providers, 1 (5%) had developed 2 apps; we did not indicate which one in order to prevent its identification.

^b^✗: no.

^c^✓: developer provided this specific reason.

^d^EEA: European Economic Area.

#### App Stores

Neither store provided information about its review process and collaboration with European regulatory authorities. Apple App Store’s guidance states that “drug dosage calculators must come from the drug manufacturer, a hospital, university, health insurance company, pharmacy or other approved entity, or receive approval by the FDA or 1 of its international counterparts” [20]*.* No such clause was found in the Google Play Store guidance [21].

## Discussion

### Principal Findings

We systematically reviewed the CE accreditation of PDCs available on 2 mobile app stores in the Netherlands. Of 74 sampled PDCs, 1 (1.4%) had the appropriate CE marking in conformity with the MDD. At a time when European regulatory authorities are seeking to enhance their scrutiny of medical apps, for example, through the MDR, this study sheds a new light on several barriers to CE accreditation for eligible mHealth apps.

This study delivered several new insights. It revealed that almost all PDCs available for download in the Netherlands fail to comply with European regulations on medical devices. The only app that is certified under the MDD (Neomate; see Table 1) will likely require additional assessment due to the more stringent classification requirements of the MDR [3,8,22]. The status quo with regard to CE accreditation for PDCs available on app stores is concerning, especially considering the fact that these apps are widely used by clinicians and have the potential to cause harm. Of the 74 PDCs identified on the screened stores, 13 (17.6%) had been downloaded over 100,000 times. Our findings thus echoed those of earlier studies highlighting the widespread use of mHealth apps among clinicians, including pediatricians [1,3].

Multiple reasons were identified for PDC manufacturers’ poor compliance with European regulations. First, PDC developers appeared to have varying levels of awareness of the existence of such regulations. For those manufacturers that knew about these regulations, European rule interpretation was ambivalent. Several developers argued that their apps do not qualify as medical devices according to the relevant European standards. This was true despite the clear statement by the MDD, the MDR, and associated European and European member state guidance that any software involving manipulation of data intended to be used for diagnostic or treatment purposes in individual patients qualify as a medical device [3,6,8,17,22]. The concept of *intent of use* seemed especially prone to a variety of interpretations by manufacturers. Many of those interviewed, as well as the end-user licenses of multiple sampled apps, indicated that their PDCs were for reference or educational purposes only. This claim, however may be in conflict with the *actual* use of such apps by their users, given their functionality. Although data on PDC usage by clinicians is scarce, anecdotal evidence suggests that the advice generated by such apps is frequently used to inform real patient care.

Reflecting on the functionality of their apps, some developers highlighted a difference between pediatric drug dosage calculators and calculators of infusion volumes or rates [23]. The difference, they contended, pertained to the type of information being input into the app and the data delivered by it, as well as the complexity and transparency of the computations it ran. Although drug dosage calculators generated medication advice based on individual patient characteristics, this was not the case for infusion rate calculators that performed conversion calculations on a pre-established drug dosage. This distinction, however, does not align with MDD guidance nor with the MDR, which take the stance that any app involving transformation of data subsequently informing the treatment of an individual patient qualifies as a medical device, irrespective of the complexity of the transformation [6,7,17].

In addition to disagreements on the substance of European law, another barrier hampering broader CE accreditation of eligible apps concerned the technical nature and potential costs associated with this process. According to the MDD and the MDR [6,8], the onus of certification falls on providers that may lack the capacity to take on the associated liability and costs [24,25]. The challenging nature of the conformity assessment process will only increase under the MDR, given the up-classification of software apps, leading to additional evaluation requirements, including the appointment of a notified body [8,9]. In this context, a general lack of institutional support for developers seeking CE accreditation for their apps may become even more discouraging.

Another factor likely to undermine the compliance of PDC manufacturers with European standards on medical devices concerns the lack of an established process for enforcing these rules at a premarket stage. As with other European legislations, the enforcement of the MDD and the MDR is incumbent upon each EU member state [26]. Although the Dutch Decision on Medical Devices states that the distribution and use of apps that fail to obtain a CE mark is forbidden [27], it does not provide any enforcement means before such apps become available on app stores. Restrictive measures are unlikely to be taken unless a medical error resulting from the use of software occurs, especially if the latter leads to litigation. In this case, the responsibility for the medical error falls on both app users and the app developer [3]. Although the MDR tightens the requirements for CE accreditation and enhances postmarket surveillance [28], it does not fundamentally change the principle by which software manufacturers are themselves responsible for initiating the CE marking process [8]. Effectively, the EU’s reliance on this framework in the absence of institutional support for developers and of control mechanisms at a premarket stage may have contributed to making other actors, for example, app stores, informally responsible for restricting European market access. It also implies that clinicians (or their institutions) wishing to use a PDC should themselves assess whether an app is properly accredited despite their lack of expertise in such matters [5].

Among other measures, these findings speak to the need for making CE marking information more readily available to PDC users. This may be achieved through the planned extension of the European Database on Medical Devices (EUDAMED), scheduled to become publicly accessible in May 2022 [29,30], and through the introduction of unique device identifiers for medical devices across the EEA, expected by 2024 [31]. EEA member states may also choose to build on existing online registries of certified or evidence-based apps [16,32]. European authorities could seek to formally engage app stores as partners in the enforcement of the European MDR. At this stage, it appears that the initiative for restricting access to app stores resides with the app stores themselves, as illustrated by the various levels of restrictions described in the guidance documents of Apple App Store and Google Play Store. The finding that more PDCs were available on Google Play Store (66/74, 89.2%) than on Apple App Store (28/74, 10.8%) may suggest that differences in the stringency of requirements contributed to developers’ decisions on where to make their apps available.

### Limitations

This work had several limitations. Web-based PDCs that did not have a mobile interface, for example, the Dutch Paediatric Formulary calculator, which was developed in conformity with the requirements of the MDD [33,34], were excluded. The restricted search functions of app stores limited the comprehensiveness of the search possible, for example, excluding paid-for apps. As a result, the list of PDCs included from those stores may not be exhaustive and may only apply to apps available for download in the Netherlands. Eight apps that were only available for purchase were excluded. Considering the potential differences between freely available apps and apps that were available for purchase and whose manufacturers may thus rely on additional finances to recover the costs associated with obtaining a CE marking, this could have led to selection bias. Despite the existence of MDD guidance stating that CE accreditation should be clearly displayed on app landing pages in the relevant stores [17] and our cross-referencing of multiple sources, it is possible that 1 or more CE-marked PDCs were misclassified. In the absence of a mandatory statement on CE accreditation on the app stores, PDC developers were contacted directly. Additionally, 2 of the 61 (3.3%) developers could not be contacted due to missing contact information, and only 20 of the 59 (33.9%) developers contacted provided responses. This relatively low response rate was likely to introduce response bias into the qualitative component of the study. We expect therefore that those developers who responded may represent those who wish to be accessible to those with questions about their apps, and as such their responses may not be representative of all app developers.

### Conclusion

This study demonstrates that almost no PDC currently available on two app stores accessed in the Netherlands adheres to European regulations on CE marking. In addition to the limited awareness of these norms among PDC developers, this compliance gap can be related to incorrect rule interpretation by some app manufacturers, the lack of mechanisms for verifying mHealth apps’ compliance with European medical device rules before market access, and the technical nature of the CE accreditation process for developers often lacking institutional support.

Although limited to a single category of apps, it is likely that these findings apply to a broader set of mobile devices being used in clinical settings. This lack of regulatory compliance puts both clinicians and patients at risk of medical errors resulting from the use of uncertified and, in some cases, potentially unsafe PDCs. This practice therefore undermines the potential impact of the MDD and the MDR, which strive to create a technologically safer European medical landscape, while supporting clinicians’ trust in the devices they use.

## Appendix

Multimedia Appendix 1 Email to developers (email body).

Multimedia Appendix 2 List of excluded apps and reasons for exclusion.

## Abbreviations

EEA: European Economic Area
EU: European Union
EUDAMED: European Database on Medical Devices
MDD: Medical Devices Directive
MDR: Medical Device Regulation
MHRA: Medicines and Healthcare products Regulatory Agency
PDC: pediatric drug calculator
PRISMA: Preferred Reporting Items for Systematic Reviews and Meta-Analyses
CE: Conformité Europénne
