# Supplementary material for: CE Accreditation and Barriers to CE Marking of Pediatric Drug Calculators for Mobile Devices: Scoping Review and Qualitative Analysis
Source: J Med Internet Res. 2021 Dec 13;23(12):e31333. doi: 10.2196/31333 (PMC8713103; doi:10.2196/31333)
Supplement: Multimedia Appendix 1 [file jmir_v23i12e31333_app1.docx]

**Multimedia Appendix 1.** Email to developers (email body).

I am a final-year medical student at Utrecht University in the Netherlands. I am currently writing a research paper identifying mobile health (mHealth) apps that include a pediatric drug calculator (PDC) and looking at the CE marking of these apps as medical devices.

I am interested in finding out whether these apps have received a classification and have performed a conformity assessment for European market access in line with the European Medical Devices Directive (MDD) and the more recent Medical Device Regulation (MDR). I am also looking at the barriers for doing so for app manufacturers.

I am writing this email to you concerning your “Infinite dose: the smart dosage calculator” app. I could not find any information about the CE marking of your app in its description on app stores or in the app itself. Would you mind answering the following questions in this regard?

1. Is your app intended to be used by clinicians in the European Union (EU)?

2. If so, in what year was it introduced in the European market?

3. Is your app CE marked?

4. Do you know its classification:

    - Under the MDD (Class I, IIa, IIb, or III)?

    - Under the MDR (Class I, IIa, Iib, or III)?

5. Has your app undergone a conformity assessment procedure (by you or a European Notified Body) for market access:

     - Based on its classification under the MDD?

     - Based on its (up)-classification under the MDR?

6. Have you encountered any barriers with regard to:

     - Interpreting the European law on the definition, classification, or conformity assessment procedures for medical devices?

     - Getting your application CE marked (certified)?

Any additional comment on this topic is welcome.
