# Supplementary material for: CE Accreditation and Barriers to CE Marking of Pediatric Drug Calculators for Mobile Devices: Scoping Review and Qualitative Analysis
Source: J Med Internet Res. 2021 Dec 13;23(12):e31333. doi: 10.2196/31333 (PMC8713103; doi:10.2196/31333)
Supplement: Multimedia Appendix 2 [file jmir_v23i12e31333_app2.docx]

**Multimedia Appendix 2.** List of excluded apps and reasons for exclusion.

| **Name of app** | **App provider** | **Reason for exclusion** |
| --- | --- | --- |
| AAP Pediatric Care Online | American Academy of Pediatrics Apps | Requires purchase |
| Anesthesia infusion calculator | Crystal Clear Solutions | Requires purchase |
| Calculate by QxMD | QxMD Medical Software Inc. | No drug calculator |
| Clinical Guidelines | The Royal Children's Hospital, Melbourne | No drug calculator |
| Coly ICU | MAGNA HEALTH SOLUTIONS | Only applicable outside the European Economic Area (EEA) |
| Critical Care Calculator | Prestaciones Médicas RCCC | Requires purchase |
| CURRENT Diagnosis and Treatment Pediatrics | Skyscape Medspresso Inc | No drug calculator |
| Davis's Drug Guide | Unbound Medicine, Inc | No calculator specifically for use in pediatrics |
| Davis’s Drug Guide for Nurses - 2020 updates | Skyscape Medpresso Inc | No calculator specifically for use in pediatrics |
| Dose Calculator | Wayne C. Johnson | No calculator specifically for use in pediatrics |
| Drug Center: Pediatric Oncall | Pediatric Oncall | No drug calculator |
| Drugs Dictionary | KURY | No drug calculator |
| Drug of Choice | M&A Developers | No drug calculator |
| Emergency Drugs | NMKG | No drug calculator |
| Emergency Nurse Practitioner | The Royal Children's Hospital, Melbourne | No drug calculator |
| EMRA Antibiotic Guide | Emergency Medicine Residents' Association | No calculator specifically for use in pediatrics |
| Gomella's Neonatology | Skyscape Medpresso Inc | Requires purchase |
| GP Antibiotics | Polwarth Medical Ltd | No drug calculator |
| Handbook of Clinical Anesthesia | Skyscape Medspresso Inc | Requires purchase |
| Harriet Lane | Unbound Medicine, Inc | No drug calculator (preview) |
| Harriet Lane Handbook App | Skyscape Medpresso Inc | No drug calculator |
| Harriet Lane Handbook Pediatric Drug | Skyscape Medpresso Inc | No drug calculator |
| Infusion And Drugs Calculator - Counting Drops | Specialslice | No calculator specifically for use in pediatrics |
| Infusion Calculator: Fluids, Dosages, Medications | iMedical Apps | No calculator specifically for use in pediatrics |
| Infusion pump | Dušan Merta | No calculator specifically for use in pediatrics |
| Infusion rate calculator | Chernyshkov Evgeny | No calculator specifically for use in pediatrics |
| Infusion rate calculator PRO | Chernyshkov Evgeny | No calculator specifically for use in pediatrics |
| IV Infusion Calculator | Jonsap | No calculator specifically for use in pediatrics |
| Medical & Drugs Dictionary | Itsoftgroup | No drug calculator |
| Medical Calculators | IOBear | No calculator specifically for use in pediatrics |
| Medical Calculators | WhichMan | Requires purchase |
| MicroGuide | Horizon Strategic Partners Ltd | Only for British NHS Trusts |
| Mosby's 2020 Nursing Drug Reference (Skidmore) | Skyscape Medpresso Inc | No calculator specifically for use in pediatrics |
| Nurse Calculator | TIPWEB MEDIA | No calculator specifically for use in pediatrics |
| Nursing Calculator | Firing Solutions | No calculator specifically for use in pediatrics |
| Nursing Calculator | Iván Oliver | No calculator specifically for use in pediatrics |
| Nursing Drug Handbook | Lippincott Williams Wilkins | No calculator specifically for use in pediatrics |
| Nursing Med - IV | Pablo Robledo Casado | No calculator specifically for use in pediatrics |
| Pediatric Formulary | UBQO Limited | No drug calculator |
| Pediatric Intensive Care | The Royal Children's Hospital, Melbourne | No drug calculator |
| Pediatric Intensive Care | Medipod Ltd | No drug calculator |
| Pediatric Support | Luis Sunol Mateo | Requires purchase |
| QuickEM | Bill Dirkes | No calculator specifically for use in pediatrics |
| Skyscape Medical Library | Skyscape Medpresso Inc | No drug calculator |
| Super Infusion Calculator | iOBear | No calculator specifically for use in pediatrics |
| Tarascon Pharmacopoeia | AtmosphereApps | Requires purchase |
